# Treatment Tactics for Patients with Isolated Injuries of the Fifth Lumbar Vertebra

**DOI:** 10.17691/stm2021.13.5.04

**Published:** 2021-10-29

**Authors:** S.V. Likhachev, V.V. Zaretskov, V.B. Arsenievich, V.V. Ostrovskij, I.N. Shchanitsyn, A.E. Shulga, S.P. Bazhanov

**Affiliations:** Senior Researcher, Department of Innovative Projects for Neurosurgery and Vertebrology, Research Institute of Traumatology, Orthopedics and Neurosurgery Saratov State Medical University named after V.I. Razumovsky, 112 Bolshaya Kazachia St., Saratov, 410012, Russia; Leading Researcher, Department of Innovative Projects for Neurosurgery and Vertebrology, Research Institute of Traumatology, Orthopedics and Neurosurgery; Professor, Traumatology and Orthopedics Department Saratov State Medical University named after V.I. Razumovsky, 112 Bolshaya Kazachia St., Saratov, 410012, Russia; Head of Trauma and Orthopedics Department No.3, Research Institute of Traumatology, Orthopedics and Neurosurgery Saratov State Medical University named after V.I. Razumovsky, 112 Bolshaya Kazachia St., Saratov, 410012, Russia; Director of the Research Institute of Traumatology, Orthopedics and Neurosurgery Saratov State Medical University named after V.I. Razumovsky, 112 Bolshaya Kazachia St., Saratov, 410012, Russia; Senior Researcher, Department of Innovative Projects for Neurosurgery and Vertebrology, Research Institute of Traumatology, Orthopedics and Neurosurgery Saratov State Medical University named after V.I. Razumovsky, 112 Bolshaya Kazachia St., Saratov, 410012, Russia; Researcher, Department of Innovative Projects for Neurosurgery and Vertebrology, Research Institute of Traumatology, Orthopedics and Neurosurgery Saratov State Medical University named after V.I. Razumovsky, 112 Bolshaya Kazachia St., Saratov, 410012, Russia; Head of the Department of Innovative Projects for Neurosurgery and Vertebrology, Research Institute of Traumatology, Orthopedics and Neurosurgery Saratov State Medical University named after V.I. Razumovsky, 112 Bolshaya Kazachia St., Saratov, 410012, Russia

**Keywords:** traumatic injury of the lumbosacral transition, L_5_ vertebra fractures, transpedicular fixation, anterior column support

## Abstract

The major management technique for lumbar burst fractures is transpedicular fixation (TPF). However, in relation to fractures of the L_5_ vertebra, this tactic often has no advantages over conservative treatment, and, therefore, it is expected to be supplemented with anterior decompression and reconstruction of the anterior column of the L_5_ vertebra.

**The aim of the study** was to determine the most optimal treatment tactics for patients with isolated burst fractures of the fifth lumbar vertebra.

**Materials and Methods:**

We performed a retrospective study of 58 patients treated for isolated burst L_5_ fractures. 12 patients refused to undergo surgery and received conservative outpatient treatment. TPF was performed in 27 patients; circular spondylosynthesis (TPF + anterior column support with a Mesh implant) — in 19 patients. The effectiveness of the treatment was assessed by clinical and introscopic research methods.

**Results:**

The radiological and functional outcomes of surgery with conventional TPF for isolated L_5_ burst fractures are generally comparable with the outcomes of conservative treatment. In 26% of the patients, the instability of the metal construction developed within 12 months after surgical intervention. Supplementing the transpedicular system with wedging anterior column support with a Mesh implant ensures preservation in 21%, and improves the parameters of the sagittal profile of the lumbosacral transition in 79% of cases.

## Introduction

Compression burst fractures of the fifth lumbar vertebra are quite rare and account for up to 1.6% of all injuries of the spinal column [1–3]. The data on injuries in this localization is limited to descriptions of a series of cases, the largest of which is represented by 14 patients [4].

To date, there is no uniform view on the tactics of treating patients with isolated injury to the L_5_ vertebra. The complexity of surgical reconstruction in this kind of trauma is due to the unique topographic, anatomical, and biomechanical features of this region. Bisegmental transpedicular fixation (TPF), which is a conventional solution for thoracolumbar trauma in the L_4_–S_1_ segments, is not recommended by some authors due to the lack of proven advantages over conservative treatment for injuries to the lumbosacral transitional spine [5–7]. The counter-argument regarding the use of this technique is its limited ability to maintain normal parameters of lumbar lordosis at the L_4_–S_1_ level. Frequently failed fusion of fractures, the development of kyphotic deformity, and disturbance of the sagittal balance are accompanied by the formation of chronic pain syndrome and a worsening quality of life. With minimal bone trauma in patients with uncomplicated spinal injuries, most authors suggest keeping to conservative treatment [8, 9]. Prevention of secondary kyphotization at the L_5_ vertebra level consists of restoring the supportability of the anterior column of the spine by performing anterior column support. This type of surgery is associated with technical difficulties due to the adherence of the great vessels to the anterior surface of the vertebral body. There are also available papers containing contradictory results of treatment of isolated patients who underwent anterior column support from the posterior access [10, 11].

The biomechanics of the lumbosacral transition is characterized by the transition of the mobile lumbar spine to the relatively immobile pelvis. The combination of shearing and compressing forces at the L_5_–S_1_ level demands increased stability of spondylosynthesis in case of an injury in this localization [12–14].

**The aim of the study** was to determine the optimal treatment tactics for patients with isolated burst fractures of the fifth lumbar vertebra.

## Materials and Methods

### General patient characteristics

The retrospective study included 58 patients (31 men and 27 women, median age 35 years) who were treated for isolated burst fractures of the L_5_ vertebra at the Research Institute of Traumatology, Orthopedics and Neurosurgery, Saratov State Medical University named after V.I. Razumovsky (Russia) between 2010 and 2020.

The fractures were morphologically classified according to the AOSpine classification system. 38 patients (65% of the cases) had been injured in a road traffic accident, in 15 cases (26%) the cause of the L_5_ vertebra injury was a fall from a height, in 5 cases (9%) it was a fall of an object with a large weight. Concomitant fractures of the bones of the upper and lower limbs occurred in 9 patients (16%). The median time from injury to surgery was 20 days. In 12 cases, the patients refused to undergo surgery and received conservative treatment (corset therapy, physiofunctional treatment) on an outpatient basis. Bisegmental TPF was performed in 27 patients, circular spondylosynthesis (TPF  + anterior column support with a Mesh implant) was performed in 19 patients.

### Surgical technique

*Bisegmental transpedicular fixation* was performed by a conventional technique, including a median access, skeletonization of the posterior structures of the L_4_–L_5_–S_1_ vertebrae, bilateral transpedicular insertion of monoaxial transpedicular screws, reclination of the L_5_ vertebral body due to distraction on the lordosis bar, arthrodesis at L_4_–L_5_ and L_5_–S_1_ levels.

At the first stage of *circular fixation*, standard TPF was performed with the installation of screws in the L_4_ and S_1_ vertebrae. Then, after placing the patient on his back, retroperitoneal access to the L_5_ vertebra and adjacent intervertebral discs was performed. The segmental vessels on the L_4_ vertebra body on the left, the left iliolumbar vein, and the median sacral artery were ligated and transected. As a rule, the L_4_–L_5_ disc was isolated by means of displacing the great vessels medially, while the L_5_–S_1_ disc was isolated between the iliac veins. This was followed by L_4_–L_5_ and L_5_–S_1_ discectomy and partial resection of the L_5_ vertebrae. Column support was performed with a Mesh implant filled with the autologous bone of the resected vertebral body, mixed with synthetic osteoinductive material.

**Figure 1.**
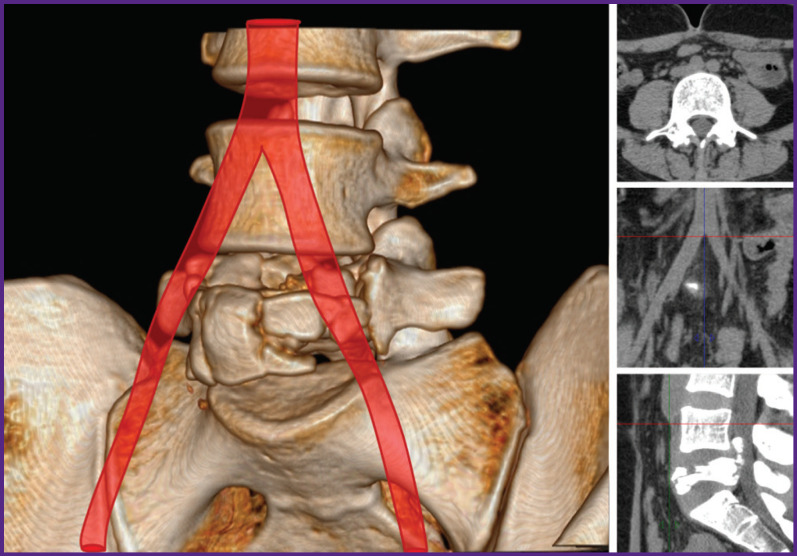
High location of the aortic bifurcation in a patient with a L_5_ vertebra fracture

**Figure 2.**
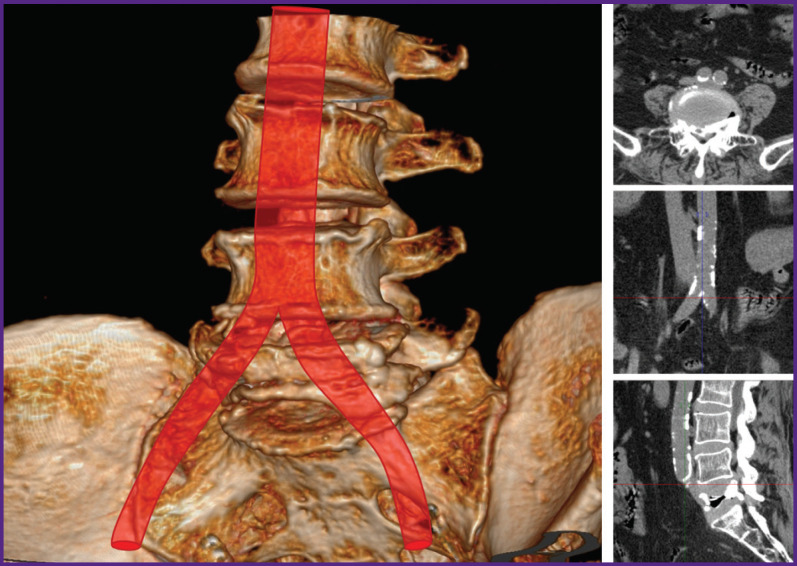
Low location of the aortic bifurcation in a patient with a L_5_ vertebra fracture

With ventral access (19 patients), the preoperative CT scan was used to determine the location of the aorta and inferior vena cava bifurcation to select the optimal access to the L_5_ vertebra. The native CT scan, in all the cases, allows for a clear determination of the aorta bifurcation, however, it is not always possible to differentiate the place of fusion of the iliac veins into the inferior vena cava, therefore, we focused on the aorta bifurcation. In most cases, the inferior vena cava was at the same level or slightly lower. In 4 patients with a high location of the aortic bifurcation (above the middle of the L_4_ vertebra) (Figure 1), the iliac arteries and veins were isolated, the access to the L_5_ vertebra was performed in the space between the iliac vessels. With low bifurcation (15 patients) (Figure 2), the left iliac arteries and veins, as well as the terminal part of the aorta and inferior vena cava were isolated with ligation of segmental vessels; the great vessels were retracted medially, and a lateral access to the L_5_ vertebra was performed. A vascular surgeon was involved in all the cases of ventral accessing.

### Introscopic research methods

All the patients underwent X-ray examination of the transitional lumbosacral spine in two projections before and after surgery (after 7 days, 2, 6, and 12 months); and CT scans of the L_4_, L_5_, S_1_ vertebrae were performed 7 days after the operation. Segmental kyphotic deformity, the height of the anterior supporting column of the spine, and the severity of the spinal lumen deficit were assessed.

Segmental deformity at the level of injury was measured according to the technique accepted for this area of the spine (the Cobb angle, that is between the tangent to the superior endplate of the L_4_ vertebra and the tangent to the superior endplate of the S_1_ vertebra [5]). The spinal lumen deficit was calculated by correlating the parameters of the deformed spinal canal at the level of injury with the literature data [15].

### Clinical research methods

The intensity of the pain syndrome was assessed using a visual analogue scale (VAS), the functional state of the patient was assessed using the Oswestry Disability Index (ODI). Intraoperative blood loss, duration of surgical interventions, and complications were recorded.

### Statistical research methods

Statistical data processing was performed using Microsoft Excel 2010 and SPSS 21.0 software (USA). To select the methods of descriptive and analytical statistics, the distribution normality was assessed using the Kolmogorov–Smirnov and Shapiro–Wilk criteria. The distribution of the majority of quantitative parameters did not correspond to normal, except for age and L_4_–S_1_ angle according to Cobb before surgery. Taking into account a small size of the sample and the abnormal distribution of most of the studied parameters, it was decided to use the methods of nonparametric statistics. The quantitative data were presented as median and quartiles, Me [Q1; Q3]; the qualitative features were taken in absolute values and percentages. The analysis of differences between the groups in terms of quantitative characteristics was carried out using the Mann–Whitney U-test (for two groups, two-sided test) and Kruskal–Wallis test (for three or more groups). For the quantitative data, in the case of related samples, the paired Wilcoxon signed-rank test (for two groups) and Friedman’s two-way ANOVA by ranks (for three or more groups) were used. Comparing the groups by qualitative features was carried out by evaluating contingency tables and calculating the χ^2^ criterion. When the number of observations in the cells of the table was less than 5, Fisher’s exact test was used (two-sided test). The differences were considered statistically significant at p<0.05 for all the methods. When significant differences were identified between the three groups after applying the Kruskal–Wallis test, a new, corrected for the number of comparisons, critical level of significance was assessed in posteriori pairwise comparisons (p<0.017; Bonferroni correction — α=0.05/3).

## Results

The general characteristics of the patient groups in the preoperative period are presented in Table 1. There were no statistically significant differences between the groups of patients in terms of body mass index, time from the moment of injury to the moment of surgery (or initial examination in case of conservative treatment), neurological status, and intensity of pain syndrome according to VAS. The statistically significant differences in the age and sex composition and the initial functional status according to ODI are noteworthy and they are apparently associated with the small size of patient groups.

**Table 1:** General characteristics of patient groups in the preoperative period

Parameter	Group 1, conservative treatment (n=12)	Group 2, TPF (n=27)	Group 3, TPF + Mesh (n=19)	р*	р**		
					1–2	1–3	2–3
Age (years)	30 [20; 37]	43 [37; 46]	32 [25; 54]	**0.045**	0.04	0.435	0.843
BMI	25 [22; 26]	25 [24; 29]	21 [25; 26]	0.314	—	—	—
Men, n (%)	9 (75)	16 (59)	6 (31)	**0.044**	0.477	0.06	0.08
Time after trauma (days)	20 [16; 24]	17 [12; 24]	20 [12; 30]	0.68	—	—	—
Neurological status (AOSpine), n (%):							
N0	12 (100)	23 (85)	12 (63)				
N1		1 (4)	3 (16)	0.112	—	—	—
N3		3 (11)	4 (21)				
VAS initially (points)	7 [6; 8]	7 [6; 8]	7 [7; 9]	0.207	—	—	—
ODI initially (%)	30 [30; 32]	34 [28; 39]	40 [38; 40]	**0.015**	0.084	**0.004**	0.122
Densitometry (Т-criterium)	1.0 [–0.7; 2.0]	1.0 [1.0; 2.0]	1.0 [–0.5; 1.0]	0.377	—	—	—
AOSpine, n (%):							
A2	0	10 (37)	0				
A3	7 (58)	5 (18)	6 (32)				
A4	5 (42)	8 (30)	13 (68)	**0.001**	**0.012**	**<0.001**	**0.002**
C	0	4 (15)	0				

Notes: for quantitative characteristics, the median and quartiles have been determined; * calculation of the χ^2^ test (Fisher’s exact test) and the Kruskal–Wallis test; ** the critical level of significance corrected for the number of comparisons (p<0.017) for pairwise comparisons.

**Figure 3.**
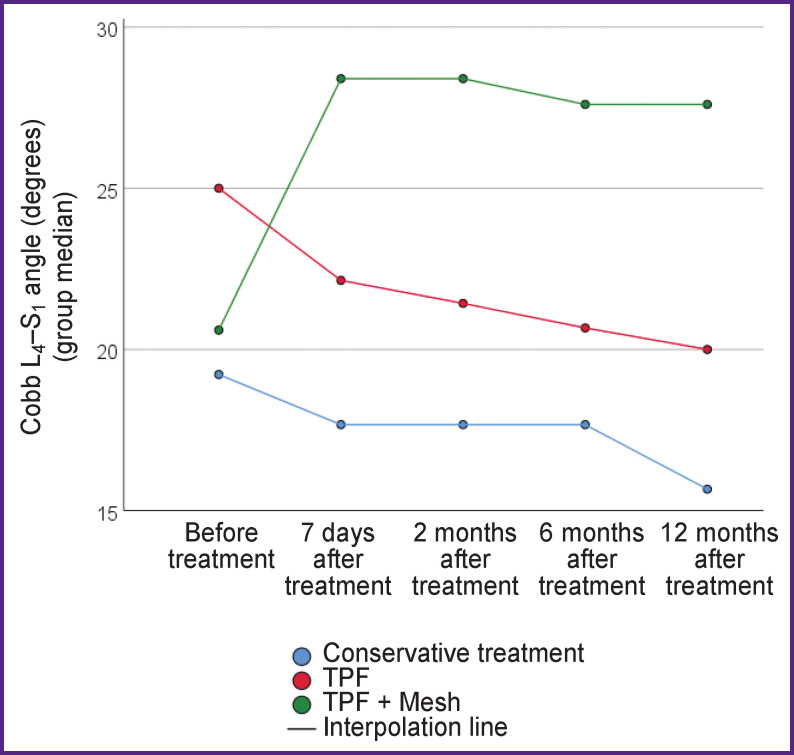
Dynamics of changes in the regional angle L_4_–S_1_ according to Cobb

**Figure 4.**
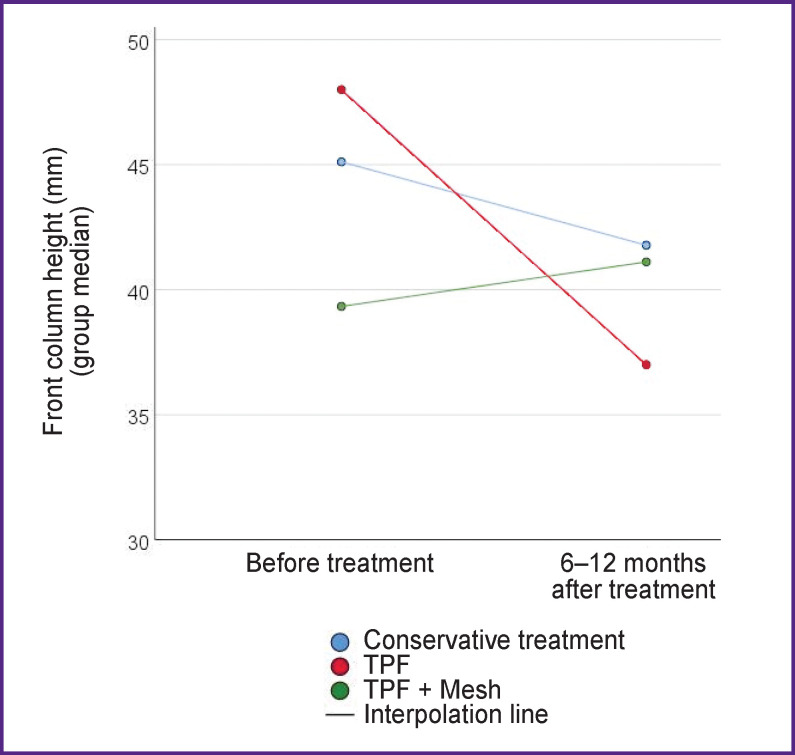
Dynamics of changes in the height of the anterior column of the L_5_ vertebra

The bulk (93%) of the patients were those with burst fractures type A. The dynamics of changes in X-ray and clinical parameters in the patient groups is presented in Table 2. Graphically, the dynamics of changes in the regional L_4_–S_1_ angle according to Cobb and the height of the anterior supporting column of the L_5_ vertebra is shown in Figures 3 and 4, respectively.

**Table 2:** Clinical and introscopic parameters of patient groups, as well as intraoperative complications

Parameter	Group 1, conservative treatment (n=12)	Group 2, TPF (n=27)	Group 3, TPF + Mesh (n=19)	р*	р**		
					1–2	1–3	2–3
L_4_–S_1_ angle by Cobb (degrees):							
before treatment	21 [13; 31]	25 [20; 31]	20 [17; 25]	0.053	0.104	0.704	0.023
7 days after treatment	19 [13; 28]	20 [19; 29]	29 [23; 31]	**0.033**	0.091	0.02	0.112
2 months after treatment	19 [13; 27]	20 [19; 29]	29 [23; 31]	**0.025**	0.111	**0.016**	0.071
6 months after treatment	19 [13; 27]	20 [17; 29]	28 [23; 30]	**0.048**	0.298	0.02	0.079
12 months after treatment	16 [13; 27]	20 [16; 29]	28 [23; 30]	**0.03**	0.221	**0.01**	0.077
р***	0.016	**<0.001**	0.002	—	—	—	—
L_4_–S_1_ angle correction after treatment, n (%)	0	5 (18)	15 (79)	**<0.001**	0.299	**<0.001**	**<0.001**
Negative dynamics by the L_4_–S_1_ angle after 12 months, n (%)	6 (50)	13 (48)	2 (11)	**0.018**	0.594	0.032	**0.01**
Spinal lumen deficit before treatment, n (%)	9 (75)	16 (59)	9 (47)	0.141	—	—	—
Correction of the spinal lumen deficit 6–12 months after treatment, n (%)	0	0	7 (78)	**<0.001**	—	**0.003**	**0.001**
Hight of the anterior column (mm):							
before treatment	46 [42; 48]	48 [34; 50]	39 [37; 50]	0.287			
6–12 months after treatment	42 [41; 45]	37 [30; 49]	40 [40; 55]	0.143			
Operation time (min)	—	115 [110; 140]	230 [210; 240]	**<0.001**	—	—	**<0.001**
Blood loss (ml)	—	200 [160; 200]	650 [600; 750]	**<0.001**	—	—	**<0.001**
Duration of hospital stay (days)	—	6 [6; 8]	7 [6; 9]	0.768	—	—	—
Instability of fixation 6–12 months							
after treatment, n (%)	0	7 (26)	1 (5)	**0.031**	—	—	0.061

Notes: for quantitative characteristics, the median and quartiles have been determined; * calculation of the χ^2^ test (Fisher’s exact test) and the Kruskal–Wallis test; ** adjusted for the number of comparisons, the critical level of significance (p<0.017) in pairwise comparisons; *** Wilcoxon test (comparison before treatment and 12 months after it).

Circular fixation (TPF  +  wedging anterior column support with a Mesh implant) has the advantage of a better reconstruction of the sagittal profile of the lumbosacral transition compared with the isolated use of TPF, which reflects the dynamics of changes in the regional Cobb angle and the height of the anterior column of the L_5_ vertebra. Supplementing TPF with a Mesh implant provides not only additional correction when the L_4_–S_1_ gap is wedged, but also ensures the maintenance of the correction achieved during the operation.

The decrease in the regional angle L_4_–S_1_ according to Cobb after intervention with TPF is noteworthy. Conservative treatment does not reduce the regional angle L_4_–S_1_ in the first months of treatment, but in the long term (12 months) leads to its greater degradation in comparison with surgical technologies. Among all the treatment methods, only circular fixation (TPF  + Mesh) and, accordingly, anterior decompression provide adequate release of the content of the spinal canal from compression by bone fragments of the damaged vertebral body.

The instability of the metal structure (fracture of the transpedicular screws in all the cases) was detected in 7 (26%) patients operated on with transpedicular fixation, and in 1 patient after circular fixation.

When using anterior access, vascular complications and damage to the superior hypogastric plexus should be considered as specific iatrogenies. Despite the fact that in all the cases with ventral access a vascular surgeon was part of the operating team, injuries to the great vessels were observed in 4 (21%) cases during isolation (the left common iliac vein in 2 cases, the inferior vena cava in the bifurcation area in one case, and the left common iliac artery on the left in one case). Vascular injury occurred in 3 cases with a median access (between the iliac vessels) and in 1 case with a lateral access (lateral from the great vessels). It should be noted that in all the cases, an adhesive process was observed around the vessels in the area of the L_5_ vertebra fracture, which occurred due to a longer time from the moment of injury (more than 21 days). The inferior vena cava was damaged when it was exposed during skeletonization of the L_5_ vertebral body. All the vascular injuries were sutured with a vascular suture while maintaining blood flow through the vessels without significant blood loss. There was no further evidence for arterial and venous thrombosis.

**Figure 5.**
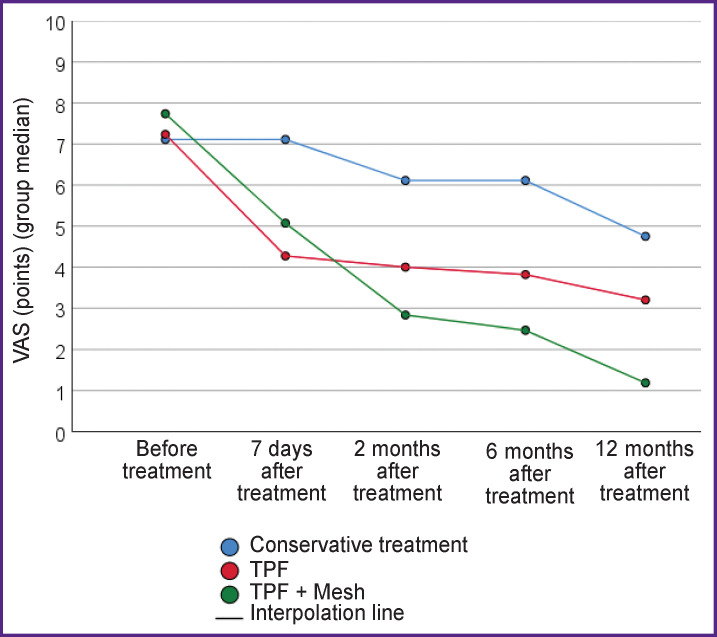
Dynamics of changes in pain syndrome according to the visual analogue scale

When comparing the clinical results of treatment (Table 3, Figure 5), more favorable (an antalgic effect and functional state) outcomes of circular fixation were revealed. Despite the high trauma of TPF + Mesh operation, a statistically significant decrease in the intensity of pain syndrome compared to the other two groups of patients has been noted during the follow-up observation.

**Table 3:** Clinical parameters of patients according to VAS and ODI

Parameter	Group 1, conservative treatment (n=12)	Group 2, TPF (n=27)	Group 3, TPF + Mesh (n=19)	р*	р**		
					1–2	1–3	2–3
VAS (points):							
before treatment	7 [6; 8]	7 [6; 8]	7 [7; 9]	0.207	—	—	—
7 days after treatment	7 [6; 8]	4 [3; 6]	5 [5; 6]	**<0.001**	**<0.001**	**0.001**	0.42
2 months after treatment	6 [5; 7]	4 [3; 5]	3 [2; 4]	**<0.001**	**0.002**	**<0.001**	0.035
6 months after treatment	6 [5; 7]	4 [3; 5]	2 [2; 3]	**<0.001**	**0.001**	**<0.001**	0.061
12 months after treatment	5 [4; 5]	3 [3; 4]	1 [1; 2]	**<0.001**	**0.006**	**<0.001**	**0.01**
р***	**0.001**	**<0.001**	**<0.001**	—	—	—	—
ODI (points):							
before treatment	30 [30; 32]	34 [28; 39]	35 [33; 40]	**0.015**	0.365	**0.012**	0.251
7 days after treatment	30 [29; 30]	28 [20; 30]	26 [24; 26]	**0.014**	0.024	0.022	0.226
2 months after treatment	28 [28; 30]	18 [18; 20]	20 [16; 20]	**0.002**	**0.003**	**0.003**	0.253
6 months after treatment	26 [26; 28]	18 [14; 18]	14 [14; 16]	**<0.001**	**0.002**	**<0.001**	0.295
12 months after treatment	26 [20; 26]	14 [10; 18]	10 [6; 10]	**<0.001**	**0.002**	**<0.001**	0.101
р***	**0.001**	**<0.001**	**<0.001**	—	—	—	—

Notes: for quantitative characteristics, the median and quartiles have been determined; * calculation of the χ^2^ test (Fisher’s exact test) and the Kruskal–Wallis test; ** adjusted for the number of comparisons, the critical level of significance (p<0.017) in pairwise comparisons; *** Wilcoxon test (comparison before treatment and 12 months after).

## Discussion

Fractures of the fifth lumbar vertebra are rare. The burst nature of the fracture, as a rule, is the consequence of application of a critical force along the axis of the spinal column, leading to the propulsion of bone fragments into the lumen of the spinal canal and the formation of kyphotic deformity. The anatomy and biomechanics of the lumbosacral junction determine the morphology of fractures, which differs from injuries to the thoracolumbar region. Burst fractures (A3, A4 according to AOSpine) of the L_5_ vertebra are more stable, in contrast to similar destruction of the thoracolumbar transitional vertebra, due to the location below the iliac crests and additional stabilization by the iliolumbar ligaments. Significant compression of the L_5_ vertebral body in some cases can lead to changes in the biomechanics of the spine due to sagittal imbalance. The combination of a large cross-section of the spinal canal at the L_5_ vertebra level and a relatively greater resistance to injury to the cauda equina roots compared to the spinal cone contributes to a small number of neurological complications in isolated burst fractures of the L_5_ vertebra [1–9].

According to a number of authors [3, 9], burst fractures are considered unstable in the presence of neurological deficit, significant loss of vertebral body height, as well as in case of kyphosis more than 20° and a deficit in the lumen of the spinal canal exceeding 40%. Optimal surgical tactics for these fractures include decompression of neural structures in neurological disorders, complete correction of the deformity, and stabilization at the level of injury.

The viewpoints regarding the treatment of patients with isolated injuries of the L_5_ vertebra are controversial. Thus, in a number of publications [4, 6, 8, 9] it is reported that the clinical and radiological results of conservative treatment and bisegmental TPF are comparable in neurologically uncomplicated fractures. In contrast, Mick et al. [16] note an unsatisfactory dynamics in the form of a decrease in the height of the anterior parts of the compressed L_5_ vertebra in patients receiving conservative treatment, contrasting it with the data of reclination of the vertebral bodies in patients who underwent transpedicular spondylosynthesis. Kaminski et al. [5] indicate that such interventions are fraught with the loss of correction in the long-term postoperative period. Despite the significant loss of correction, the functional results, as a rule, do not correlate with the introscopic ones. The effect of decompression of the content of the spinal canal is also not obvious in relation to the spontaneous regression of radiculopathy [17].

**Figure 6.**
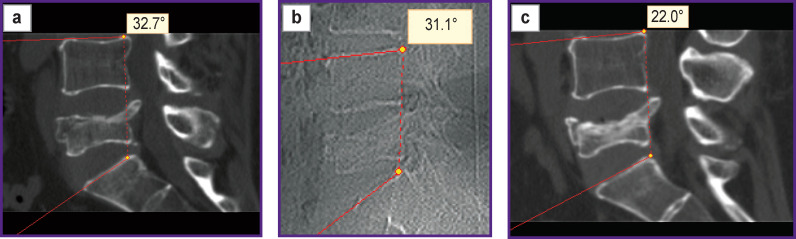
CT scan and X-ray examination of patient R., 45 years old: (a) 10 days after injury; (b) 6 months after the start of conservative treatment; (c) 12 months after the start of conservative treatment. Attention should be paid to the stabilization of the L_4_–S_1_ regional angle from the beginning of the follow-up to 6 months and its progressive decrease by 12 months

A decrease in the regional angle L_4_–S_1_ after implantation of the transpedicular system, accompanied by a deterioration in the quality of life, and less pronounced regression of pain in the long-term period compared with circular surgical reconstruction, does not give reasons for recommending this variant of spondylosynthesis as a method of choice. A decrease in the regional angle L_4_–S_1_ is likely to be associated with the distraction of the dorsal parts of the spinal motion segments L_4_–S_1_ while the reclining effect on the anterior supporting column of the L_5_ vertebra being insufficient. With conservative treatment, better indicators of the regional angle L_4_–S_1_ are observed up to 6 months after injury compared with TPF due to a uniform decrease in the height of the anterior and middle supporting columns in the absence of implanted instrumentation. However, no radiological or clinical benefits of conservative treatment are observed after 12 months (Figure 6).

**Figure 7.**
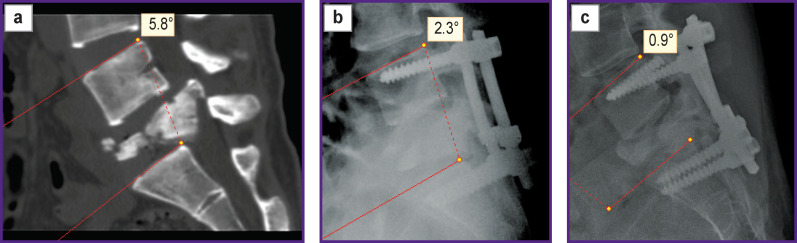
CT scan and X-ray examination of patient R., 35 years old: (a) 10 days after injury; (b) 6 months after transpedicular fixation; (c) 12 months after transpedicular fixation. Attention should be paid to a decrease in the L_4_–S_1_ regional angle in the postoperative period up to 6 months. Instability of the metal structure developed 12 months after surgery

With bisegmental TPF, a number of patients with the L_5_ vertebra burst fracture (A4) developed instability of the metal structure by 12 months after surgery (Figure 7).

The clinical efficiency of surgical reconstruction for injuries of the L_5_ vertebra can be increased by supplementing TPF with anterior column support in combination (if necessary) with decompression of the spinal canal contents. The experience of using Mesh implants in the reconstruction of the resected vertebral body at the level of the thoracolumbar transitional spine [18–20] can be extrapolated to the lumbosacral transition. The practice of circular fixation and decompression in burst fractures of the L_5_ vertebra is not sufficiently presented in the available literature [1, 21]. Transpedicular spondylosynthesis of the lumbosacral transitional spine is not stable enough to minimize the risk of developing instability of the metal structure and recollapse of the vertebral body.

**Figure 8.**
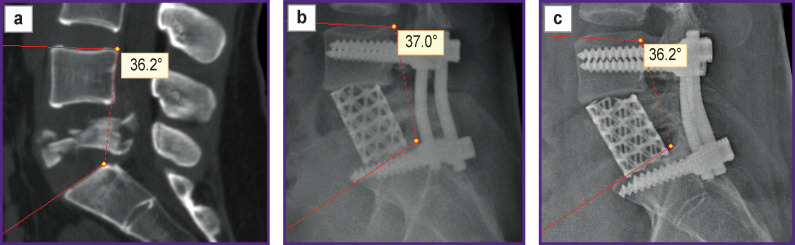
CT scan and X-ray examination of patient P., 29 years old: (a) 7 days after injury; (b) 7 days after transpedicular fixation and anterior column support with a Mesh; (c) 12 months after transpedicular fixation and anterior column support with a Mesh

**Figure 9.**
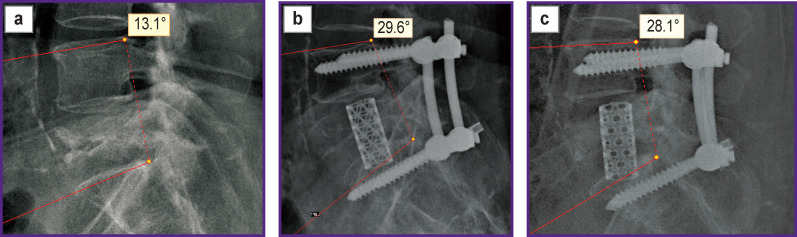
CT scan and X-ray examination of patient M., 53 years old: (a) 7 days after injury; (b) 7 days after transpedicular fixation and anterior column support with a Mesh; (c) 12 months after transpedicular fixation and anterior column support with a Mesh

Taking into consideration the dominant role of the L_4_–L_5_ and L_5_–S_1_ segments in the formation of lumbar lordosis, it can be argued that, in addition to restoring the height of the L_5_ vertebra, it is necessary to reconstruct and ensure the preservation of the harmonious sagittal profile of the spine at this level. This should contribute to the normalization and maintenance of normal sagittal balance of the spine. Such outcomes can be achieved by a combination of the transpedicular system and anterior column support with a Mesh implant (Figures 8 and 9).

Normal lordosis at the L_4_–S_1_ level is about 40° [22]. Injury leading to L_5_ deformity alters the sagittal balance of the patient’s spine. Although its long-term consequences are not widely described in the literature, disturbances in the sagittal balance in patients with deformities and degenerative-dystrophic lesions of the spine correlate with a decrease in the quality of life [23]. With regard to the L_5_ vertebra in this study, the results correlated with the previously reported data [1], since the functional results being satisfactory, an improvement in the regional angle L_4_–S_1_ was noted.

**Study limitation**. The study contains limitations associated with its retrospective design, the inability to assess the sagittal balance due to the lack of images in a “standing” position in the majority of patients before surgery and a small number of subjects. It should be noted that the data were obtained for the period from 2010 to 2020. The surgical technology and instrumentation have progressed considerably since then. This provided a radical approach to the reconstruction of the L_5_ vertebra by expanding the possibilities of resection of the L_5_ vertebral body and performing the L_4_–S_1_ wedging anterior column support. Thus, today, isolated TPF can be considered as an adequate method of treatment for A2 and C types of injuries. Circular spondylosynthesis provides better outcomes in burst fractures (A3, A4).

## Conclusion

Radiological and functional outcomes of using conventional transpedicular fixation in isolated burst fractures of the L_5_ vertebra are comparable with the outcomes of conservative treatment and do not contribute to the achievement of patient-satisfying parameters. This is most likely associated with the negative effect of transpedicular fixation of the L_5_ vertebra in fractures of types A3 and A4, since due to distraction of the dorsal regions, lordosis decreases at the L_4_–S_1_ level compared to the preoperative characteristics. Such results can be explained by the anatomical features of the lumbosacral transitional area (its location below the iliac crests and additional stabilization by the iliolumbar ligaments). In our study, the isolated use of transpedicular fixation was associated with the development of instability of the metal structure in 26% of cases within 12 months. Supplementing the transpedicular system with a wedging anterior column support of the Mesh provides preservation in 21% of the cases and improves the parameters of the sagittal profile of the lumbosacral transition in 79% of the cases. The involvement of a vascular surgeon is advisable when performing anterior column support at this level.
